# Medical fears of the malingering soldier: ‘phony cronies’ and the Repat in 1960s Australia

**DOI:** 10.1017/mdh.2023.19

**Published:** 2023-04

**Authors:** Effie Karageorgos

**Affiliations:** School of Humanities, Creative Industries and Social Sciences, The University of Newcastle, University Drive, CALLAGHAN NSW 2308, Australia

**Keywords:** Trauma, repatriation, psychiatry, malingering, soldiers, Medicine

## Abstract

The fear of the malingering soldier or veteran has existed in Australia since its first nationwide military venture in South Africa. The establishment of the Repatriation Department in 1917 saw the medical, military and political fields work collectively, to some extent, to support hundreds of thousands of men who returned from their military service wounded or ill. Over the next decades the medical profession occasionally criticised the Repatriation Department’s alleged laxness towards soldier recipients of military pensions, particularly those with less visible war-related psychiatric conditions. In 1963 this reached a crescendo when a group of Australian doctors drew battle lines in the correspondence pages of the *Medical Journal of Australia*, accusing the Repatriation Department of directing a ‘national scandal’, and provoking responses by both the Minister for Repatriation and the Chairman of the War Pensions Assessment Appeal Tribunal. Although this controversy and its aftermath does allow for closer investigation of the inner workings of the Repatriation Department, the words of the doctors themselves about ‘phony cronies’, ‘deadbeats’ and ‘drongoes’ also reveal how the medical fear of the malingering soldier, and particularly the traumatised soldier-malingerer, lingered into the early 1960s and beyond. This paper will analyse the medical conceptualisation of the traumatised soldier in the 1960s in relation to historical conceptions of malingering, the increasingly tenuous position of psychiatry, as well as the socio-medical ‘sick role’, and will explore possible links with the current soldier and veteran suicide crisis in Australia.

In the 1960s, psychiatry as a medical specialty experienced increasing instability and faced challenges not only from the medical establishment, but also anti-psychiatrists and social activists.^1^ Focussed on the reorientation in social worlds from the nineteenth century, anti-psychiatrists each created slightly differing models that largely placed responsibility upon the state and its need for social control for the determination of ‘the boundary between deviant and respectable’.^2^ Within these models, mental illness was frequently redefined as ‘deviance’ to an extent decided by relevant social authorities, and the mentally ill were ‘labelled’ as such to protect members of society who conform to its rules.^3^ Some anti-psychiatrists went so far as to deny the existence of ‘mental illness’, including the Hungarian-American psychiatrist Thomas Szasz in his 1961 book *The Myth of Mental Illness.* ^4^ These ideas appealed to the growing activist movement, particularly disability activists who supported a less punitive approach to the mentally ill. Many also insisted on a repositioning of mental illness from the medical model to the social model, which was influential in the Australian shift to social psychiatry. ^5^

Ideas about deviance within considerations of illness did not originate in the anti-psychiatry movement, however. In 1951 Talcott Parsons wrote his key text *The Social System*, in which he raised the existence of a ‘social contract’ that requires humans to continually work towards and conform to societal norms.^6^ Within this social contract, he highlighted the ‘sick role’, which in his words was ‘one of the most important withdrawal behaviours in our society’.^7^ Although the ‘sick role’ has limited applicability today, due to its outdated gendering, as well as key changes in the structure of world societies and approaches to health care, it was an influential concept within the sociology of medicine until the 1980s.^8^ John Burnham writes that the social behaviours outlined in the ‘sick role’ were accepted and recognised by medical professionals because of their pertinence to mid-twentieth century western societies.^9^

Parsons views illness as a form of deviance determined by both medical knowledge and social norms and attributes four specific aspects to the ‘sick role’:

1. The sick person must be excluded from ‘normal social role responsibilities’, decided upon by the physician.
2. The sick person ‘cannot be expected by “pulling himself together” to get well by an act of decision or will’.
3. The sick person must want to get well.
4. The sick person must seek ‘technically competent’ help from a physician and ‘cooperate’ with them so as to get well.^10^

This article centres on a collection of letters that appeared in the *Medical Journal of Australia* (*MJA*), written by physicians treating returned soldiers in the 1960s, which was followed by *Be In It, Mate!*, a 1969 book written by John Whiting, a doctor working in a South Australian repatriation hospital. Parsons’ framing of the ‘sick person’ as someone with no control over their illness and the necessary primacy of physicians in providing cure was consistently reflected in the repeated questioning of the legitimacy of psychiatric claims of repatriation benefits by the ‘phony crony’, or malingering veteran, within these writings. For example, ‘Parkinson’s Second Disciple’ describes ‘due to war service psychiatric cases’ in a letter to the *MJA* on 12 October 1963:

The laymen may imagine that these are poor folk who have been mentally deranged through horrifying war experiences. Most of us know better. The majority are just the dead-beats, drongos, drunkards and inadequate members of society who are encouraged by the Department to seek shelter from their responsibilities in its wards, and higher and higher pensions to perpetuate their way of life.^11^

The doctors discussed within this article were evidently influenced by their assigned role since the First World War as arbiters of repatriation policy and investigators of soldier malingering, motivating them to publicise their views about Australian repatriation policy.^12^ The idea of the malingering soldier, and malingering in general, was also increasingly addressed within the *MJA*, as well as in popular fiction, from the Second World War, meaning that these ideas would have been prominent in the minds of physicians working with returned soldiers.^13^ Detection of the ‘phony crony’ was not an issue specific to Australia, as McNally highlights the persistence of what he labels the ‘phony combat vet’ in the United States from the Vietnam period, suggesting that malingering and specifically the presence of ‘phony’ repatriation cases has been a continuous issue over the twentieth century.^14^ The adoption of Parsons’ ‘sick role’ by the mid-twentieth century medical establishment suggests that this theory offers a useful frame for analysing the allegations of malingering that appeared in these texts, directed by Australian physicians towards pensioned traumatised veterans – most of whom had fought in the Second World War and Korea – and particularly the apparent significance of alcoholism or drunkenness as an assumed facet of the mentally ill veteran experience.^15^

Pensioned mentally ill veterans seemingly transgressed their socially ordained role as an ill person in need of medical help in a range of ways, some legitimate and others merely due to perceptions by physicians about mental illness and psychiatry. This transgression means that the specialist knowledge of physicians in treating sickness was rendered inadequate, leading to frustration and thus rejection by physicians.^16^ This element – combined with the effects of factors including historical perceptions of malingering, which Goldberg confirms has consistently integrated bias related to broader ideas about race, gender, disability and class – as well as the increasingly tenuous position of psychiatry within society contributed to a specific rendering of the mentally ill veteran by Australian doctors.^17^ An investigation of the ways that Parsons’ ideas related to the ‘sick role’ appeared within discussions of medicine, psychiatry and particularly soldier or veteran health in 1960s Australia allows for a closer exploration of the specific reasons for medical rejection of the traumatised veteran and the reliance on malingering as an alternative explanation for the seemingly ambiguous symptoms of war trauma. In addition, while this study focuses specifically on the Australian context, the ideas explored within it are applicable to veterans across the world, as the involvement of the medical establishment in the pursuit of soldier-malingerers, as well as the widespread critique of psychiatry, have occurred globally. This article thus uses a range of Australian and international historical, sociological and psychological perspectives on mental illness, war trauma and malingering to explore the medical fear of the traumatised ‘soldier-malingerer’ in 1960s Australia.

## The Repat doctors and the *Medical Journal of Australia*

On 3 August 1963, a letter of condemnation directed at the Repatriation Department appeared in the correspondence section of the *MJA.* The author, who used the pseudonym ‘Parkinson’s Disciple’, began the letter by asserting that ‘The Repatriation Department appears to be trying to prove one of Parkinson’s theories, that Government departments once created must make more and more work for themselves in order to justify their existence’.^18^ The letter’s author urged the Australian Medical Association (AMA) to take a ‘critical look at repatriation medicine’, claiming that ‘too much is being spent on the “phonies”’, and presenting examples of veterans receiving pensions for either non-existent medical conditions or illnesses unconnected with their military service.^19^ The letter ended by urging the Australian Federal Government to ‘insist on stricter control of allotment of entitlements, and even have the right of veto in some cases’, while noting that ‘more could and should be done’ for those with ‘genuine claims to Repatriation benefits’. The letter also attracted the attention of *The Canberra Times*, which published an article, also on 3 August, titled ‘Doctor Attacks Department’, summarising the thoughts of Parkinson’s Disciple.

This media attention prompted the serving Minister for Repatriation, Reginald Swartz, and the Chairman of the 6^th^ War Pensions Assessment Appeal Tribunal, Kevin Mooney, to write letters in response, published in the 24 August 1963 issue of the *MJA.* Swartz’s response was relatively balanced, undoubtedly keeping in mind continuing criticism of the Repatriation Department (hereafter Repat) by both the Returned & Services League of Australia (RSL) and the newspaper *Smith’s Weekly* for excessive harshness towards veterans applying for pensions.^20^ He pointed out that physicians themselves decided on the medical status of a veteran, and that the Repatriation System was specifically designed to ‘ensure that no legitimate claim is rejected’ and that the Repat was determined to ‘give the claimant the benefit of any doubt’.^21^ Mooney was more direct in his letter, labelling Parkinson’s Disciple ‘inappropriate’ and ‘unkind’ and merely ‘considers he should not be called out from his warm bed to treat ex-servicemen’, claiming that if he felt he was underpaid for his work with veterans, he should write to the Repat to have his name removed from their list of approved physicians.^22^

This did not end the matter, as Parkinson’s Disciple responded more frankly on 21 September 1963 about the ‘phony cronies … absorbing far too much of the Department’s time, money and resources’, which led to a rush of letters from a range of physicians working with Australian veterans both within Repatriation hospitals and in private practice who agreed in part or in full with the original sentiments.^23^ In these letters a variety of allegations were made, from Robert S. Lawson writing that veterans were given ‘Commonwealth cars’ to see medical specialists on Saturday mornings, then ‘find their own way to the football on Saturday afternoon’; to ‘Parkinson’s Second Disciple’ labelling the Repat ‘preposterous’, asserting that they were perpetuating a ‘national scandal’ by ‘contributing large sums to keep a bunch of happy scroungers, unscrupulous rogues and inadequate specimens’.^24^ Also in response to Swartz and Mooney’s statements, a group of South Australian doctors wrote a letter directly to Swartz to complain about the ‘medical inanities’ within the repatriation system and request an amendment of the *Repatriation Act*, which was signed by more than sixty per cent of the doctors working at the one repatriation hospital.^25^ Although Swartz did not acknowledge their letter, the Repatriation Commission Chairman Sir Frederick Chilton wrote a ‘secret letter’ to the doctors to reject all allegations sent, which was read to them by the State Deputy Commissioner.^26^

The issue appeared to be resolved from the Repat’s perspective, despite a few similar letters appearing in the *MJA* in 1966, until the mass resignation in 1969 by most who had signed the 1963 letter. This included John Whiting, a young medical officer in the Repatriation General Hospital at Daw Park, Adelaide, who in the same year published *Be In It, Mate!*, his fictional account of the experiences of ‘Dr Andrews’ – presumably based on himself –within a Repatriation hospital. The foreword to *Be In It, Mate!* states that ‘although the characters are fictitious, this story is based on fact. The author uses this medium to expose the misuse of taxpayers’ money perpetrated by politicians, public servants and others’.^27^ Whiting felt that he was ‘a pawn in “a huge extravagant political hoax”’, ending his book with a detailed Appendix stating the recent events, from the *MJA* correspondence onwards. The controversy this created within the Repat eventually led to a series of internal investigations, culminating in the *Independent Enquiry into the Repatriation System*, a report led by Justice P.B. Toose, in 1975.^28^

Significant reforms were prompted by these events that improved the workings of the Repat, all of which fall outside the scope of this article. These have all been very well outlined by Clem Lloyd and Jacqui Rees, as well as by Stephen Garton.^29^ Instead, this article examines exactly how Australian doctors construed the mentally ill veteran and positioned veterans within this ‘national scandal’. The terms of this construction initially appeared within the original letter of August 1963 written by Parkinson’s Disciple: ‘There is a whisper that alcoholism is to be an accepted disability in any psychiatric case. One shudders at the consequences of this.’^30^ On 12 October, Parkinson’s Second Disciple wrote, in addition to the above quote, that ‘the most scandalous of all…are the so-called “due to war service psychiatric cases”’.^31^ In Whiting’s *Be In It, Mate!*, the fictional Dr Andrews expressed his view of psychiatric patients by initially stating: ‘Why persist in giving it this grandiose title of ‘Psychiatric Wing’, when you know as well as we do that it’s overflowing with just plain drunks and no-hopers.’^32^ Later, after learning he would be sent to ‘Malingerer’s Mansion’, Andrews responded ‘you mean they’re sending me to the Psychiatric Ward?’^33^ After beginning work on the ward, Andrews described the psychiatric cases in discussion with his father:

I’ll tell you what our psychiatric cases consist of; about five per cent are genuinely sick fellows with some mental derangement. Nearly all of those would have been deranged whether there had been a war or not. About ninety per cent are rather hopeless ineffectuals who can’t adjust themselves to the complexities of a modern society; and of these, about three quarters are just plain drunks, bums and no-hopers.^34^

Within these and many other similar sentiments about mentally ill veterans in the *MJA* letters and *Be In It Mate!*, three recurrent themes appear. These are allegations of malingering; comments about degeneracy; and alcohol, drunkenness or alcoholism in connection to those pensioned for psychiatric disorders. To understand why these specific themes appeared within doctors’ representations of mentally ill veterans, it is necessary to consider historical and theoretical influences on physicians during this period. While society was becoming more informed about mental illness and more accepting of the mentally ill, physicians continued to express condescending views of the traumatised veteran. Although these views can be seen as a consequence of the historical conceptualisation of malingering, and specifically the historical role of physicians in its identification, this disdain can be seen to run deeper.^35^

## Attitudes towards malingering in Australia

Malingering is by no means merely a historical issue. The American military psychologist Kenneth Morel wrote in 2010 that: ‘In military medicine the detection of malingering is a necessary function of mental health practitioners.’^36^ Recent psychological literature still addresses the issue of malingering, and a range of scientific models have been formulated to detect it.^37^ In addition, Goldberg highlights key similarities between political and military concerns about malingering within United States social welfare policy from the nineteenth century until today.^38^ The *Diagnostic and Statistical Manual of Mental Disorders* (DSM-5-TR) defines malingering as ‘the intentional production of false or grossly exaggerated physical or psychological symptoms, motivated by external incentives such as avoiding military duty, avoiding work, obtaining financial compensation, evading criminal prosecution, or obtaining drugs’.^39^ The approach of the American Psychological Association towards malingering since it was first included in the DSM-III in 1980 was to explain it in terms of ‘unconscious and pathological’ motive.^40^ The move towards the official pathologising of malingering can be seen to have partly originated in medical responses to malingering during the First World War.

Although malingering has been a concern of Australian military-medical personnel since the South African War, it was not until the First World War that medical officers on the battlefield were specifically instructed to detect it.^41^ Early twentieth century British doctors were reluctant to engage in this new role, so they transformed malingering from a criminal to a medical diagnosis, meaning they could attribute malingering to heredity or psychological unfitness and thus allow them to ‘keep their medical hat on’.^42^ The medicalisation of malingering also occurred within the Ottoman Army during the conflict. The creation of the ‘pathological malingerer’ similarly allowed medical officers to disregard every negative influence on the malingerer, including reasons for avoidance of military duty and criminal responsibility, and focus on medical cure.^43^ Parson’s concept of the ‘sick role’ assumes that the sick person must be ‘helpless and therefore in need of help’, and the role of physicians are thus to serve as a ‘court of appeal as well as a direct legitimizing agent’ in confirming the ‘sickness’ of the patient and using their specialist medical knowledge to help them get well.^44^ Detecting criminality in the form of malingering falls outside the medical role, so doctors moved towards redefining the malingerer as a patient to preserve the rules surrounding their prescribed role.

Australian medical officers on the First World War battlefield also felt ‘uneasy’ about adopting ‘judicial and political functions’ in detecting malingering, so they actively moved to reframe the malingerer, but most did so by emphasising aspects of psychological unfitness that originated from weakness and a lack of morality rather than the actual processes of the mind.^45^ Military psychiatry was not an predominant concern of the Australian military forces until the First World War, whereas Britain had established a hospital for traumatised soldiers and veterans in 1869.^46^ During the First World War, the British Royal Army Medical Corps began invaliding men with shell shock in 1914, whereas the Australian Imperial Force – despite fighting alongside British forces in many cases – only began in 1916.^47^ Australian medical officers believed that malingering was the ‘action of the morally weak’ and linked it to heredity or ‘social infirmity’.^48^ This is not to say that British military-medical authorities did not also attribute psychological disorders to the ‘will’ or that all Australian medical officers moved away from psychology and towards social issues in explaining the large numbers of traumatised men.^49^ In fact, the Victorian psychiatrist John Springthorpe actively used the teachings of Sigmund Freud in treating shell shocked Australians from the years of the conflict.^50^ When assessing reasons for ‘nervous’ or mental symptoms, Australian medical officers were merely more likely to focus on the morality and social life of a soldier.

Over the course of the First World War, British doctors were more likely to move away from conceptualising shell shock and other war-related traumatic disorders in terms of heredity, moving towards organicist or psychological explanations, or a combination of both. These more ‘sympathetic’ responses to mentally ill soldiers and veterans that separated their symptoms from their personality or lifestyle did not continue into later wars, however.^51^ In the Australian context, military authorities during the Second World War remained more reluctant to engage with military psychiatry than Allied nations, despite the lessons of the First World War. In the early 1940s, William S. Dawson, Professor of Psychiatry at the University of Sydney, listed a series of ‘warning signs’ for the ‘recognition of psychopathic types’ in the military, which included specifically social characteristics such as ‘untidiness, lack of cleanliness, homesickness’ and those who were ‘indefatigable scribes and diarists’.^52^ While American psychiatrists believed that war neurosis was predominantly caused by ‘exhaustion and fatigue’ in the 1940s, Australians attributed it to poor social relations and general weakness.^53^

The greater attention to personal characteristics and lifestyle in the assessment of war neurosis is reflected in the sentiments shared publicly by the Australian Repat doctors, who in the 1960s were predominantly treating veterans of the world wars and Korea. For example, a letter by ‘Parkinson’s Apostle’ published in the *MJA* on 30 May 1964 claimed men with psychiatric entitlements were not mentally ill, but instead ‘a group of inadequate individuals who would have broken down under the day-to-day stress of almost any walk of life’.^54^ It is possible to see these attitudes as a continuation of the construction of the traumatised soldier-malingerer that originated during the First World War. However, this was not the only influence on the opinions of Repat doctors in the 1960s towards the mentally ill veteran.

## Mental illness in 1960s Australia

The decades after the Second World War saw widespread shifts in the attitude of general populations across the world towards the mentally ill. From the 1950s until the 1970s, a range of psychological, psychiatric and medical studies were carried out, many in Britain and the United States but also in other countries including India and Australia, to determine how people felt about psychiatry, mental illness and the mentally ill. Many of these were directed towards the general population, but also towards medical students, military officers and physicians. Studies directed towards students were aimed at assessing the decreasing popularity of psychiatry as an area of university study from the 1960s onwards, which occurred partly as a result of debates between advocates of the medical and social models of mental illness, to be discussed later in this article.^55^ Many of these studies used the Opinions about Mental Illness Scale (OMI) developed by Cohen and Struening, which scored respondents based on five criteria: authoritarianism; benevolence; mental hygiene ideology; social restrictiveness; and interpersonal aetiology. However, other models were also developed, such as the Custodial Mental Illness Ideology (CMI), the California F Scale and later versions of the OMI.^56^ Interestingly, most studies of the general public, military officers or physicians found a higher level than expected of benevolence or generally sympathetic views towards the mentally ill, although this changed when the suggestion was made that ‘sanity’ and mental illness existed on a continuum, meaning that anybody could become ‘insane’.^57^

The OMI scale was formulated in response to specific views towards mental illness and psychiatry emerging in the 1960s, including the move away from a medical model to a social model in the conceptualisation of mental illness. The third criterion within the scale, ‘mental hygiene ideology’, measures the extent to which a person ascribed to the medical model by asking whether a ‘mental illness is an illness like any other’.^58^ A similar Australian study was not conducted until 1979, when Robert Kirkby and Annie James used the OMI scale on a sample of 50 Australian medical practitioners. That study found that Australian doctors scored highly on benevolence and mental hygiene, reflecting their sympathy with the mentally ill and agreement with the medical model. Their scores on authoritarianism and social restrictiveness were low, meaning that they did not generally believe that ‘the mentally ill are different or inferior’ or ‘a threat to society’.^59^ However, the authors also noted that there can be a disadvantage to high scores on mental hygiene ideology, meaning that commitment to the medical model of mental illness may mean physicians will approach mental illness the same way they approach somatic illnesses, by treating the symptoms over the causes.^60^

The framing of the research questions by Kirkby and James reflects the gradual popularity of the social model of mental illness both in Australia and across the world throughout the mid-twentieth century. The work of anti-psychiatrists throughout the 1960s, which reinterpreted and questioned medical domination over the historical treatment and care of the mentally ill, influenced this model. Foucault, for example, claimed that ‘social mechanisms’ rooted in Enlightenment ideas governed the differing processes of confinement within nations since the seventeenth century, creating ‘new social norms required for social integration’ with the purpose of creating ‘a new dividing line’ between ‘the acceptable and the blameworthy’.^61^ Other anti-psychiatrists such as Goffman and Szasz focussed on the social and cultural imperatives of the state in determining the conceptualisation of the insane.^62^ During this period people from a range of social, political and intellectual groups ‘were united in their belief that psychiatry was not a legitimate medical specialty, but one devoted to protecting its authority and enforcing societal norms associated with an unjust society’.^63^

A central tenet of anti-psychiatry is deviance theory, which interprets illness as a type of deviant behaviour, or ‘motivated deviance which results in disturbed social relations’.^64^ Parsons asserted that a ‘sick person’ was not responsible for their own deviance, due to the four attributes mentioned above, namely, that the sick person must be excluded from ‘normal social roles’; cannot get well as a result of their own will; must want to get well; and must work with a physician to get well.^65^ Anti-psychiatrists drew upon Parsons’ conception of deviance to further define psychiatric symptoms as ‘violations of a social norm’. Thomas J. Scheff, a key anti-psychiatrist, introduced the term ‘residual deviance’ to describe ‘the diverse kinds of deviation for which our society provides no explicit label’, which can lead to the label of ‘mentally ill’.^66^ Interestingly, Scheff specifically used an example drawn from military psychiatry to illustrate that residual deviance is ‘denied’ or is transitory. He used evidence from military studies that claim combat neurosis could be self-terminated if a soldier remained with their primary military unit and only given minimal medical treatment.^67^ Findings like this align with the opinion by the Australian Repat doctors in the 1960s that the morale of soldiers and veterans was reduced by ‘condonement and pensions’, or the more encouraged they were to seek medical help, and were characteristic of military views that warned against encouraging malingering by ‘coddling’.^68^

Deviance theory was also influential in Australian circles in the 1970s, although it received only a fraction of the public attention that it enjoyed in the United States and Europe, and those who considered it appeared to tend towards ‘social psychiatry’, which combined elements of deviance theory and the medical model of mental illness.^69^ In 1975, Erica M. Bates from the Department of Health Administration at the University of New South Wales surveyed a random sample of 1000 people in Sydney, as well as psychiatric professionals, clergymen, university students, nurses and business managers, to determine their alignment with the medical or social/sociological models of mental illness. Although she found that most respondents in the sample accepted the medical model, Bates concluded that the ‘medical-socio-educational model’ was more effective. Her support for the social model of mental illness was apparent throughout the article, which was seemingly based on the premise that ‘there is some danger in allowing the status and power of medicine to assume control over such a large number of deviant people’.^70^ Bates continuously emphasised her agreement with the social model of mental illness throughout the article, also writing: ‘At present, the definition of deviance tends to be tailored so that the medical profession becomes the main arbiter in what probably should be a general social problem.’^71^

Such ideas were used to criticise traditional psychiatry. This was not a new phenomenon, as it had experienced disapproval from patients, the media and the medical profession as a whole since the nineteenth century. From the First World War, however, the increased popularity of psychoanalysis, which reached its height in the 1940s and 1950s, contributed to the move away from biological conceptions of mental illness and towards psychoanalytic or psychodynamic views.^72^ The treatment of psychiatric illness by specialists without specific medical training increased the vulnerability of psychiatry to attacks by supporters of anti-psychiatry, as well as the medical profession as a whole, from the 1960s.^73^ Rabkin wrote in 1972 that mental health professionals were increasingly acknowledging that ‘mental illness can be understood as an exaggeration of particular behaviours common to all men, brought about by stressful life conditions and resulting in impairment of the ability to cope with social expectations and standards’.^74^ The medical model that viewed psychiatric symptoms as a sign of physical illness, which had existed since the late nineteenth century, was first challenged by the psychoanalytical view of mental illness as a defence against internal processes. Following this, the popularity of psychoanalytic or psychodynamic explanations for psychiatric symptoms over biological psychiatry from the Second World War shifted again in the 1960s, due to increasing disillusionment with psychoanalysis, to ‘defining symptoms as a way of dealing with external events or other people’.^75^

The attacks in the 1960s influenced by the ideas of anti-psychiatrists therefore assumed a new character that was also shaped by changing thinking about appropriate sites for treatment of the mentally ill and technological advancements that introduced new drug therapies.^76^ The experiences of the Second World War had revealed that community treatment of the mentally ill was more effective than institutional treatment.^77^ This – combined with the influence of psychoanalytic ideas based on patients’ social lives and new available drug treatments – increased the viability of ‘social psychiatry’, or allowing the formerly institutionalised to live their lives in the wider community. Dain connected the shifts in the approach towards the mentally ill during this period with the broader international protest movement emerging in the 1960s, which had as its general aim the liberation of oppressed groups. He wrote that the increased popularity of community mental health initiatives in the United States as well as the ideas of anti-psychiatrists appealed to disability activists and others who viewed institutions as an instrument of subjugation and control.^78^ Similarly, Crossley wrote that the popularity of anti-psychiatry in Britain was partly furthered by the 1960s emerging counterculture, pointing out that that British disability activist journals including *Asylums* and *PROMPT* (Protection of the Rights of Mental Patients in Therapy) frequently spoke positively about the writings of anti-psychiatrists.^79^

Such ideas were also influential in Australian medical, intellectual and activist circles. Until the 1960s Australian society held predominantly unsympathetic views towards the mentally ill and was sceptical of psychiatry and psychiatrists.^80^ Psychiatry itself was languishing, remaining seemingly attached to the continuation of the mental hospital and medical model of mental illness while experiencing challenges by progressives who espoused social psychiatry or anti-psychiatry, as well as the rights of the mentally ill.^81^ The general pattern of deinstitutionalisation in line with the social psychiatry model began in Australia in the 1960s. Between 1965 and 1975, the number of beds in Australian mental hospitals declined from 12 400 to 7400.^82^ In Australia, like elsewhere, the shift to community mental health was appealing to social activists, who increasingly spoke out against Australia’s health care system.^83^ From a political perspective, this was seen as a financially appealing way to treat the mentally ill. However, Dunlop and Pols believe that the release of patients from Australia’s mental hospitals occurred prematurely, before adequate community facilities were established to support the mentally ill outside the institution.^84^

Social psychiatry was the subject of a number of *MJA* articles from the 1950s to 1980s, mostly significantly in a 26 November 1966 article by E. Cunningham Dax titled ‘Psychiatry and Society’, which laid out his justification and plan for the rise of social psychiatry in Australia. Dax’s position as Chairman of the Mental Hygiene Authority of Victoria and support for social psychiatry, outlined in his 1961 key work *Asylum to Community*, meant that he played a prominent role in the shift from institutional to community psychiatry not only in Victoria, but also nationally.^85^ He focused on possible family or social issues such as overindulgence of children, or housing, loneliness and poverty affecting the aged that caused patients to develop psychiatric symptoms, writing that ‘many social agencies and institutions will be actively involved with mental well-being’.^86^ His support for social psychiatry was clear: ‘It would be unfortunate if, with the drugs at our disposal and the social services being developed, we tended too readily, or even officiously, to interfere in the normal adjustment to life’s everyday crises.’^87^ Despite the slow development of community mental health initiatives, the introduction of Medibank and the Community Health Program by the Whitlam government in 1973 meant that over 700 community health projects had received federal funding by 1976.^88^

The atmosphere surrounding psychiatry in the 1960s and 1970s, then, was combative. Psychiatry was under attack by anti-psychiatrists who thought their area of specialisation was repressive, particularly so within Australia, as psychiatrists appeared to cling to the medical model of mental illness and the continued espousal of institutionalisation.^89^ The growing activist movement found commonalities with the concerns of anti-psychiatrists, and a growing number of intellectuals and medical professionals supported a social model of mental illness and the Australia-wide adoption of social psychiatry.^90^ As a result, psychiatrists began to publicly seek to stake their claim on, or cement their place within, their area of medical specialisation, usually by emphasising the medical nature of mental illness and the appropriateness of medical treatments for the mentally ill.^91^ Although psychiatry was a specialisation taken within a university medical degree, Australian psychiatry was ‘divorced from the mainstream of medicine’ and ‘there existed a gulf between psychiatry and medicine which meant that a move from one sphere to the other could not unreasonably be seen as the contemporary equivalent of interplanetary travel’.^92^

## ‘The sick role’

The tendency by Repat doctors to exclude most traumatised soldiers from the body of legitimate pensioners from the 1960s reflects the historical relationship between physicians, psychiatrists and the military. Although war neurosis is mentioned within their discussion of the soldier-malingerer, the terms they used to describe the traumatised veteran: ‘dead-beats, drongos, drunkards and inadequate members of society’ are notably hostile.^93^ Doctors negotiated their rejection of the soldier-malingerer on medical grounds, as mentioned above. They found a way to redefine the investigation of malingering from a legal process to a medical one and redefine the malingering soldier from a criminal to a sick person.^94^ They were able to do this by assigning characteristics to the malingerer that ensured they fit into the specific social classification of a ‘sick person’.^95^ The traumatised soldier-malingerer, however, was not so easily categorised. The remainder of this article will analyse, using Parson’s conception of the ‘sick role’, how traumatised veterans transgressed this role in the eyes of the Repat doctors and how this led to their identification as ‘unscrupulous rogues’.^96^

The first aspect of the ‘sick role’, according to Parsons, places the judgement about whether or not the sick person should be excluded from ‘normal social role responsibilities’ on the doctor, as ‘court of appeal as well as a direct legitimizing agent’.^97^ Ultimately, then, it is the doctor’s decision whether or not the sick person is deserving of the ‘sick role’. Parsons goes on to say that ‘this legitimation has the social function of protection against “malingering”’.^98^ In most cases, he writes, this is a ‘perfectly straightforward technological job’, but for some cases ‘knowledge, skills and resources are not adequate, with hard, competent work, to solve the problem’. He labels these ‘absolute limits of the physician’s control’, which are reliant on the state of medical knowledge at the time, as a source of frustration and strain.^99^ The doctor’s role, then, is relatively straightforward, expressed well by the Australian physician C. Gordon Harper in his contribution to the *MJA* correspondence in 1963: ‘The function of the healing profession is to promote the recovery and well-being of sick people.’*^100^* In his statement supporting a medical model of psychiatry published in the *Journal of the American Medical Association* in 1975, Arnold M. Ludwig, from the Department of Psychiatry at the University of Kentucky, similarly wrote:

What distinguishes the medical model from nonmedical models…is not so much its reliance on scientific methods but rather a philosophical orientation toward dealing with symptoms and signs of patients…that sufficient deviation from normal represents *disease*, that the disease is due to known or unknown *natural causes*, and that elimination of these causes will result in *cure* or improvement in individual patients’ [original emphasis].^101^

The presence of ‘disease’, ‘natural causes’ and ‘cure’, then, seem essential for a medical professional, which points to a reason for the disdain expressed by the Repat doctors towards the traumatised veteran.

Although Australian military forces had encountered war trauma since the South African War and formally recognised it from the First World War, military authorities appeared reluctant to engage with psychiatry from the Second World War onwards, as mentioned earlier. Rather than fully engaging with psychiatry to treat traumatised soldiers and veterans, they were more likely to focus on morality and heredity in explaining symptoms.^102^ Even today physicians and mental health professionals disagree about the necessary approach to war trauma and mental illness in general, despite the comprehensive nature of the *Diagnostic and Statistical Manual of Mental Disorders.* ^103^ This means that selecting the appropriate course of treatment is not always a clear decision, and cure is not always possible. In the 1960s, the rise of psychotherapy demonstrated that mental illness could be successfully treated by nonmedical experts, which increased the ‘gulf between psychiatry and medicine’ as physicians grew increasingly sceptical of the applicability of technical medical knowledge to non-somatic conditions.^104^ Around this same time, sociologists were pointing out the existing uncertainty about the causes of mental illness within the body of medical knowledge.^105^ This appeared to confirm existing doubts from within the medical establishment about psychiatry and the applicability of medical expertise to mental illness.

The centrality of ‘cure’ to the Repat doctors is evident in both the *MJA* correspondence and in *Be In It, Mate!* The original *MJA* letter of August 1963 by Parkinson’s Disciple alleged that ‘The [Repatriation] Department does not consider that anything can be cured if one wore a uniform’, illustrating the belief that the Repat’s aims did not align with those of the Repat doctors – namely, treatment towards cure.^106^ On 5 October 1963, a letter by C. Gordon Harper of Port Macquarie was published in *MJA* in which he wrote: ‘Only once have I seen a repatriation patient cured of anything. In that case it happened only when it dawned on him that his domestic life was becoming endangered by his continual invalidism and ineffectiveness as a husband. Today he is completely rehabilitated.’^107^ Whiting also wrote in *Be In It, Mate!*: ‘The present system hardly encourages a man to admit improvement, let alone cure, if he thereby suffers a loss of pension. Our best medical efforts are thereby frustrated.’^108^ These statements demonstrate clearly the frustration described by Parsons and felt by these medical men at their inevitable failure to effect cure using their specialist medical knowledge.^109^ This does not appear to be an issue specific to this context, however. In his 1987 article ‘When Doctors Get Sick’, Spiro writes that ‘men and women are more than their bodies’, urging fellow physicians to reduce their focus on ‘final answers’, or cure, and that ‘talking about problems of life and practice, to find solutions which may not lie in the cell, could enlarge our perspective’.^110^ If medical knowledge is not entirely applicable to mental illness, then Parsons’ first aspect of the ‘sick role’ cannot be met, namely, that physicians need to use their own specialist knowledge to confirm that the sick person should be excluded from ‘normal social role responsibilities’.

Parsons’ second aspect of the ‘sick role’ again relies on the medical practitioner’s expert opinion to determine that the sick person ‘cannot be expected by “pulling himself together” to get well by an act of decision or will’.^111^ As mentioned earlier, *Be In It, Mate!* provides a description of the typical ‘psychiatric case’:

About five per cent are genuinely sick fellows with some mental derangement. Nearly all of these would have been deranged whether there had been a war or not. About ninety per cent are rather hopeless ineffectuals who can’t adjust themselves to the complexities of a modern society; and of these, about three quarters are just plain drunks, bums and no-hopers.^112^

Two themes stand out within this passage, which are also found in responses by other Repat doctors. The first is the rejection of war trauma as a genuine consequence of combat or military membership. It has long been acknowledged that military involvement can cause neurosis even in the absence of direct combat.^113^ The tendency of military authorities from the Second World War onwards to discount psychiatric explanations for mental health conditions in favour of morality or personal characteristics, as well as the existing tension between psychiatry and medicine, created an atmosphere of doubt among physicians treating traumatised veterans, as mentioned above.

The second is the relationship between heredity and war trauma, which governed medical and psychiatric considerations of such conditions until after the Vietnam War. Alistair Thomson writes that the Repat files after the First World War were notable for their ‘moral judgements about family traits and mental weakness’.^114^ Despite the increasing incidence of war neurosis in the Second World War and beyond, it was not until the publication in 1980 of DSM-III that post-traumatic stress disorder was introduced as a war-related psychiatric condition that, for the first time, was not related to heredity.^115^ This was due largely to pressure from anti-war psychiatrists such as Robert Jay Lifton and Chaim Shatan, who treated Vietnam veterans after the war.^116^

The belief that most ‘psychiatric cases’ are ‘drunks, bums and no-hopers’ also assumes that they are in control of their symptoms and illness. Whiting also wrote, through his young Dr Andrews: ‘It might be a good idea to stop molly-coddling this type of person, and get him standing on his own two feet, instead of encouraging him, as the Department does now, to lounge around and be a waster, by paying him bigger and bigger pensions’.^117^ ‘Standing on his own two feet’ implies that the veteran should be able to recover from their illness using their own ‘decision or will’, in Parsons’ words.^118^ It is evident by these statements that the Repat doctors considered ‘psychiatric cases’ to also transgress Parsons’ second aspect of the ‘sick role’.

In his 1963 *MJA* letter, C. Gordon Harper continued to compare the traumatised soldier-malingerer to ‘the normal patient who come to us with the expectation of something being done to get them off the sick list’, who ‘make an effort to get well, and stay well, to the best of one’s ability’.^119^ These align with the two remaining aspects of Parsons’ ‘sick role’ theory: that the sick person must want to get well, and that they must seek ‘technically competent’ help from a physician and ‘cooperate’ with them to get well.^120^ Most Repat doctors blamed the Repatriation Department for creating an atmosphere in which former soldiers were encouraged to ‘hang on at all costs to every listed disability or complaint, with a determination which in the case of less sheltered patients would have been sufficient to effect a cure’.^121^ ‘Parkinson’s Second Disciple’ mentioned that malingering veterans were ‘aided and abetted by bureaucracy’.^122^ Whiting wrote that Repat ‘bureaucrats, aided and abetted by certain notoriety-seeking politicians and ex-servicemen’s organizations, especially the R.S.L., are actively encouraging men and women in huge droves to come in for their pickings…most politicians, for fear of losing votes, are frightened to use the scalpel’.^123^ In the *MJA* on 30 May 1964, ‘Parkinson’s Apostle’ also wrote that ‘the present attitude of condonement and pensions only fosters them in their inadequacy and provides sufficient money for an escape into alcoholism’.^124^

The recurring topic of degeneracy in the Repat doctors’ descriptions of the traumatised soldier-malingerer, specifically related to alcohol or alcoholism, is not an unusual response by physicians. Historical accounts of malingering, especially those in relation to mentally ill soldiers, frequently demonstrated that military-medical officers attached a class judgement onto the malingerer or patient, occasionally related to cultural factors. British authorities in the mid-nineteenth century thought both civilian and soldier malingering to be a problem restricted to the working classes or foreigners. Kanaan and Wessely attribute this to the difficulty in distinguishing illnesses using clinical or psychiatric means in comparison with the ease of categorising them according to gender or class.^125^ Before the shell shock phenomenon challenged the practice of distinguishing ‘nervous’ conditions in this way, British medical officers labelled trauma experienced by officers and rank and file soldiers differently. Officers were diagnosed with ‘neurasthenia’, a more ‘respectable’ illness that had traditionally been associated with the working man, whereas rank and file soldiers were more likely to be labelled ‘hysterics’, reflecting the perceived similarities between traumatic conditions emerging from the First World War and the late nineteenth century ‘hysteria’ diagnosis, which was traditionally seen as a ‘feminine’ disease.^126^ The behaviour of traumatised Indian soldiers in the British Army was similarly categorised by British authorities in terms of ethnic and religious background, as part of a larger effort to identify social groups within India that were considered more worthy of recruitment.^127^ Medico-cultural constructions of war trauma have thus formed an essential component of the consideration of traumatised veterans by military doctors, illustrating Rosenberg’s argument about the underlying moral character of diagnosis in which he labels ‘disease entities…indisputable social actors, real inasmuch as we have believed in them and acted individually and collectively on those beliefs’.^128^ The accounts of traumatised Australian war pensioners by the Repat doctors are notable, however, due to the recurring references to alcohol.

The Repat doctors frequently tied the ‘psychiatric case’ with the ‘drunk’ or ‘alcoholic’. This includes Parkinson’s Apostle’s words above that connect the availability of military pensions with the turn to alcoholism, as well as the original *MJA* letter by Parkinson’s Disciple warning that alcoholism was to be considered a pensionable condition.^129^ The relationship between alcohol and the soldier experience has a long history. The presence of alcohol on the battle front has been labelled ‘therapeutic’, as it allows soldiers to endure the emotionally difficult aspects of the war experience, and aids in forming bonds within military units.^130^ Australian soldiers in all wars have been allowed small amounts of alcohol, and the Vietnam War was notable for the presence of ‘boozers’ on the war front, which soldiers frequently visited.^131^ The presence of alcoholism within forms of war neurosis has also been long acknowledged. Alcoholism was a common symptom of war neurosis after the First World War, and by 1939, 4891 veterans were receiving treatment for psychiatric conditions in Repatriation hospitals, some specifically for alcoholism.^132^ Numerous recent studies specifically focus on the connection between trauma in war and alcohol use, in an attempt to reduce reliance by veterans.^133^ Despite the psychological basis for certain instances of alcoholism, the tendency of Australian military-medical authorities to frame trauma in a medico-cultural way since the First World War meant that the possibility of alcohol as a symptom of a psychiatric condition was dismissed. In line with Kanaan and Wessely’s findings mentioned above, it was easier for medical professionals in search of a definable illness and achievable cure to focus on alcoholism as a personality flaw over any pathological reasons for addiction.^134^

This tendency gave rise to negative comments about alcoholic soldiers such as those cited in this article, as well as three other notable examples from *Be In It, Mate!* In one section of the book Dr Andrews’ colleague Dr Bell describes the case of a 22-year-old veteran, Mr Davidson, who:

…went A.W.L [from the hospital] a couple of months ago, got nice and pickled down at the pub, and staggered back four hours later…I kicked him out, but he’s been back twice since – came back to save up some more money for another drinking spree…Quite a war record our Mr Davidson had too – six and a half months in the Army in Victoria – deserted – was picked up two years later.

When Andrews asked whether Davidson was employed, Bell said: ‘Work! He hasn’t worked for three years – war nerves, old man. His wife is the one who goes out to work. He contributes his war pension – or what’s left of it after a week on the grog.’^135^ This passage not only connects trauma and alcoholism, it also raises Davidson’s failure to act as breadwinner in his family unit, relying on his wife to support him. Also, importantly, it mentions Davidson’s desertion from his military unit. During the First World War, the British Army initially executed ‘malingerers’ who deserted – many of whom were suffering from shell shock or another psychiatric condition – before the morality of the death penalty for such acts began to be more widely questioned.^136^ The British Army defined malingering at this time as an ‘evasion of man’s duty to the state’ and to his fellow soldiers, and desertion as its ‘most dangerous form’ was a sign of a man’s morality.^137^

In an earlier passage, Dr Andrews asked Dr Bell how many ‘real’ psychiatric cases there were in the hospital, to which Bell responded:

Only three…a paranoid, a schizophrenic and a manic-depressive. The rest are the usual Repat. Collection…a mighty crumby crowd – mainly drunks and parasites – a couple of decent ones among them – just a bit weak in the knees, that’s all…The rest are mainly old incorrigibles who will never be cured as long as the Repat continues to pay them pensions for being drunks.^138^

In the final example, Andrews responded ‘mockingly’ after a colleague, Cranford, used the word ‘drunk’ to describe Repat ‘psychiatric cases’: ‘Careful…you mustn’t use that word – drunk. You mustn’t call them alcoholics either. Only the common people are alcoholics and get drunk. A recent ruling from above says that our drunks are all nerve cases, due to the war – of course’.^139^

These passages not only interconnect malingering, alcoholism and ‘war nerves’, but also suggest that veterans who drink can be compared to alcoholics in society. An examination of medical attitudes to alcoholics and alcoholism, then, could reveal more about how the Repat doctors viewed the traumatised veteran in the 1960s and 1970s. Few Australian studies specifically investigated medical views of alcohol and alcoholism; however, there is some indication that a tension existed at the time between social and medical perspectives of alcoholism. In her 1968 article ‘Heavy Drinking and its Relation to Alcoholism’, sociologist Margaret J. Sargent, perhaps unsurprisingly, focused solely on social and cultural factors that influenced Australians who drink.^140^ The psychiatric view, however, called for greater medicalisation of alcoholism. In 1961, H. M. Southwood, President of the Australasian Association of Psychiatrists, sought to define ‘mental health’ from a psychiatric perspective in his *MJA* article ‘The Psychiatrist and the Public’. He argued against attaching social or moralistic definitions to ‘mental ill-health’, including oversimplified statements relating ‘mental sickness’ with ‘delinquency, alcoholism, broken homes, twisted lives, crime, apathy, dejection, melancholy and suicide’, writing that the psychiatric view is more complex.^141^ In his 1971 article ‘Alcoholism and Drug Dependence: Planning a New Service’, psychiatrist B. L. Hennessy discussed the importance of a medical treatment centre for alcoholics, citing both social and psychiatric factors in the development of alcoholism and labelling the lack of available psychiatric treatment ‘perturbing’.^142^

International studies prove more valuable in revealing public and medical attitudes towards alcohol and alcoholics. In 1971, American sociologists Chalfant and Kurtz analysed assessments of alcoholics by social workers in line with Parsons’ ‘sick role’, writing that more recent studies emerging from the 1950s had moved away from ‘deviance’ as an individual fault and towards definitions placed upon the ‘deviant’ by others in society. They confirmed, however, that although society was moving from a moral to medical approach towards alcoholism, individual medical practitioners still frequently blamed the alcoholic for their condition.^143^ Similarly, Abram and McCourt wrote in 1964 that despite efforts by the American Medical Association and the World Health Organisation to medicalise alcoholism, ‘At the clinical level…alcoholism continues to be viewed with the ambivalence, often expressed in feelings of derision, disgust and anger, such as were accorded other mental illnesses a generation ago’.^144^ Casper suggests a reason for this ambivalence in his study of ‘punch-drunk slugnuts’, or people with brain damage caused by head injuries who were categorised by doctors and the general public as ‘losers’, living on the fringes of society. The medicalisation of their condition was resisted by physicians, partly due to the difficulty in reconceptualising a long-standing social stigma, which Casper wrote ‘shows the material working power of culture, custom and conceit in shaping clinical knowledge’.^145^

Such feelings are illustrated by the title of some studies of the time, namely, ‘Doctors and Dirty Work’ and ‘Normal Rubbish’.^146^ In these studies, many doctors and other medical professionals expressed a sense of futility when discussing alcoholic patients that emerged from their inability to adequately diagnose and cure alcoholism:

What the hell’s the use of knowing what you’ve got in your practice, or how to deal with it, or how to diagnose if they can’t cure it - and they can’t. And I’m sure that even the best of psychiatrists will tell you that they get cures but they don’t know why they cure some and not others.^147^

If medical professionals are not able to appropriately legitimise an illness, by recognising and following a clear path to cure, Parson’s first aspect of the ‘sick role’ cannot be met, as mentioned above.^148^ Jeffery found that this led to frustration that was projected towards the patient: ‘Staff felt uncertain about the existence of an illness if there was no therapy that they, or anyone else, could provide to correct the state, and it would seem that this uncertainty fostered frustration which was vented as hostility towards these patients.’^149^ This ‘hostility’ took the form of labelling alcoholic patients as ‘rubbish’, which frequently appeared as sarcastic notes on patients’ record cards. This tendency is also supported by Braslow’s research on the treatment of neurosyphilis in the first half of the twentieth century, which found that open disdain of ‘silly’, ‘obscene’ and ‘vulgar’ patients by physicians transformed into a more respectful relationship once cure became possible through malaria fever therapy in the 1920s.^150^ Moreover, like the class judgements placed on malingerers or traumatised soldiers and veterans, socioeconomic class and cultural background were found by Mendelson *et al*. to significantly influence physician attitudes towards alcoholic patients.^151^ Also, the more senior the position of hospital staff, the more critical they were of alcoholics and drug addicts.^152^

Similar frustration emerged when alcoholic patients failed to work at their sobriety in cooperation with the doctor. Parsons’ third and fourth aspects of the ‘sick role’ require the patient to want to get well and to cooperate with physicians to do so.^153^ Mendelson *et al*. observed in their 1964 study that one of the main reasons physicians found alcoholics difficult to treat is because they ‘seldom follow [their] advice’.^154^ One of the doctors interviewed in Jeffery’s study highlighted the importance of cooperation:

I also like some patient relationships, providing the patient is a co-operative, pleasant, useful human being. I am afraid I get very short, very annoyed, with neurotic patients and with patients who I think are just drop-outs from society really – it’s a horrible thing to say – not worth helping.^155^

Physicians in these studies shared the opinion of Repat doctors that patients needed to take personal responsibility for their recovery and avoid becoming – in the words of ‘Parkinson’s Second Disciple’ in the *MJA:* ‘dead-beats, drongos, drunkards and inadequate members of society who are encouraged by the Department to seek shelter from their responsibilities in its wards’.^156^

In his 1979 study of ‘normal rubbish’ in hospitals, Jeffery found that if a physician did not hold the knowledge required to treat a patient, they may have been more likely to question the legitimacy of their illness, or their status as a ‘sick person’. A doctor interviewed in his study, when asked what type of patient was worth treating, said: ‘Anyone who is genuinely ill – I’m not talking about the psychiatric types I suppose, they’re genuinely ill but the thing is I don’t really understand psychiatric illness.’^157^ The de-medicalisation process by physicians who felt unable to work with a patient to effect a cure described by Jeffery can be directly compared to the – often furious and harsh – de-medicalisation of pensioned traumatised veterans by the Repat doctors, who were strengthened in their convictions not only by their long-term roles as detectives against malingerers, but also by the continuing ambivalence within the medical establishment and society with regard to psychiatry and psychiatrists.

## Conclusions

This article has drawn attention to the medical rendering of the ‘mentally ill veteran’ in 1960s Australia by considering historical perceptions of the malingering soldier, the increasingly tenuous position of psychiatry within society and the medical establishment, as well as the alleged transgression by these veterans of their social role as ‘sick people’ in need of medical treatment, using Parson’s ‘sick role’ concept. The consequences of sending traumatised men to be treated by medical professionals who hold such attitudes towards mental illness, and particularly mental illness arising in the hyper-masculine figure of the Australian soldier, has not yet been comprehensively analysed. It is known, however, that stigma rooted in twentieth century perceptions of soldiering still means that many soldiers and veterans do not seek help for mental health issues, contributing to high suicide rates.^158^

The events described in this article occurred just as Australian soldiers began to return from the Vietnam War with what will, from 1980, be formally recognised as post-traumatic stress disorder (PTSD).^159^ Despite continued advances in the medical, psychiatric, public, military and political knowledge of war-related trauma from the 1960s onwards, at the time of writing Australia is in the midst of a soldier and veteran suicide crisis, which is being addressed by a Royal Commission into Defence and Veteran Suicide, established on 8 July 2021.^160^ Roundtable discussions carried out thus far within the Royal Commission’s investigations have found that ‘under-reporting and hiding of…mental health issues’ have occurred because ‘medical classification was used as a means of removing people from service’. They have also found that ‘this system of official and unofficial sanctions or consequences for being perceived as weak has resulted in a widespread culture of fear in the ADF [Australian Defence Force]’.^161^ The Royal Commission’s Interim Report, delivered on 11 August 2022, similarly reports a culture of silencing, or as one submission reveals, echoing the *MJA* letters and *Be In It, Mate!*:

Recruits are often tormented if they are injured…they are often told to ‘suck it up’, ‘build a bridge and get over it’, ‘stop being a woos’ and many other demeaning or derogatory sayings. This often leads to the Recruit not attending sick parade to rectify what might on the surface be a minor issue but is something that can come back in later life as a major problem but because it wasn’t reported in the first instance the injury claim is denied because there was no record of it lodged on the person’s medical file.^162^

The culture reported in the Royal Commission’s findings thus far, which appears to be sanctioned by medical professionals working within the military, reflect some of the attitudes about ‘weakness’ described in this article. If soldiers today fear seeking help for mental health issues due to the possibility of medical punishments emerging from a disdain of ‘weakness’, it is likely that veterans suffering from war-related psychiatric conditions in the 1960s did the same when faced with medical professionals who labelled them ‘drunks, bums and no-hopers’.^163^

## Footnotes

1

Robyn Dunlop and Hans Pols , ‘Deinstitutionalisation and Mental Health Activism in Australia: Emerging Voices of Individuals with Lived Experience of Severe Mental Distress, 1975–1985’, History Australia, 19, 1 (2022), 92–114; Gerald N. Grob , ‘The Attack of Psychiatric Legitimacy in the 1960s: Rhetoric and Reality’, Journal of the History of the Behavioral Sciences, 47, 4 (2011), 398–416 2196883210.1002/jhbs.20518; Erica M. Bates , ‘Models of Mental Disorder: an Australian Survey’, Social Science and Medicine, 9 (1975), 549–51121602010.1016/0037-7856(75)90111-0; Nick Crossley , ‘R.D. Laing and the British Anti-Psychiatry Movement: a Socio-Historical Analysis’, Social Science & Medicine, 47, 7 (1998), 877–89972210810.1016/s0277-9536(98)00147-6.

2

Michel Foucault , History of Madness (Abingdon, Oxon: Routledge, 2006), 78, 82; see also Erving Goffman , Asylums: Essays on the Social Situation of Mental Patients and Other Inmates (Harmondsworth: Penguin, 1987), 4 ; R.D. Laing , The Divided Self: an Existential Study in Sanity and Madness (Harmondsworth: Penguin, 1965); Thomas J. Scheff , ‘The Role of the Mentally Ill and the Dynamics of Mental Disorder: a Research Framework’, Sociometry, 25, 4 (1963), 436–53.

3 Scheff, *ibid.,* 439; Kai T. Erikson , ‘Notes on the Sociology of Deviance’, Social Problems, 9, 4 (1962), 308 ; Bates, *op. cit.* (note 1).

4

Thomas S. Szasz , The Myth of Mental Illness: Foundations of a Theory of Personal Conduct (New York: Harper & Row, 1974), 48–9.

5 Dunlop and Pols, *op. cit.* (note 1), 92–114; Bates, *op. cit.* (note 1), 549–51; Shirley S. Angrist , ‘Mental Illness and Deviant Behaviour: unresolved Conceptual Problems’, The Sociological Quarterly, 7, 4 (1966), 136–448 .

6

Talcott Parsons , The Social System [1951] (London: Routledge, 1991), 430 .

7
*Ibid.,* 31.

8 See John C. Burnham , ‘Why Sociologists Abandoned the Sick Role Concept’, History of the Human Sciences, 27, 1 (2014), 70–87 ; Matthias Zick Varul , ‘Talcott Parsons, the Sick Role and Chronic Illness’, Body & Society, 16, 2 (2010), 73 .

9 Burnham, *op. cit.* (note 8), 71.

10 Parsons, *op. cit.* (note 6), 436–7.

11 Parkinson’s Second Disciple, ‘Repatriation Entitlements’, *Medical Journal of Australia,* 2, 15 (1963), 638.

12

Michael Tyquin , Madness and the Military: Australia’s Experience of the Great War (Loftus: Australian Military History Publications, 2006), 35 .

13 See R.J.D. Turnbull, ‘The Problems of an Army Medical Examiner’, *Medical Journal of Australia,* 2 August 1941, 111–4; Neville Parker, ‘Malingering’, *Medical Journal of Australia,* 2 December 1972, 1308–11; Williams Forrest, ‘The Girl and the Malingerer’, *Australian Women’s Weekly*, 23 March 1946, 11; Fairlee Apperly, ‘Malingerer’, *Westerly: A Quarterly Review*, 3 (1968), 41.

14

Richard J. McNally , ‘Progress and Controversy in the Study of Posttraumatic Stress Disorder’, Annual Review of Psychology, 54 (2003), 234 10.1146/annurev.psych.54.101601.14511212172002.

15 Parsons, *op. cit.* (note 6), 428–51.

16 Parsons, *op. cit.* (note 6), 447–9.

17

Daniel S. Goldberg , ‘Introduction: Investigating Malingering and Public Policy Through an Interdisciplinary Working Group’, The Journal of Law, Medicine & Ethics, 49 (2021), 339–4210.1017/jme.2021.5234665087.

18 Parkinson’s Disciple, ‘Repatriation Entitlements’, *Medical Journal of Australia*, 2, 5 (1963), 208.

19
*Ibid.*, 209.

20

Stephen Garton , The Cost of War: Australians Return (Oxford: Oxford University Press, 1996), 88–9; See also, eg., ‘Brusque Repat Brush-Off’, *Smiths’ Weekly*, 28 July 1945, 13; ‘Another Repat Blunder: Death was the Proof’, *Smiths Weekly*, 17 May 1947, 5; Commonwealth, *Lack of Confidence Motion*, House of Representatives, 14 August 1968, 150–79.

21

Reginald Swartz , ‘Repatriation Entitlements’, Medical Journal of Australia, 2, 8 (1963), 335 14044362.

22

W. Kevin Mooney , ‘Repatriation Entitlements’, Medical Journal of Australia, 2, 8 (1963), 336 14044362.

23 Parkinson’s Disciple, ‘Repatriation Entitlements’, *Medical Journal of Australia*, 2, 12 (1963), 515.

24

Robert J. Lawson , ‘Repatriation Entitlements’, Medical Journal of Australia, 2, 13 (1963), 559 14044362; Parkinson’s Second Disciple, *op.cit.* (note 11), 638.

25

Clem Lloyd and Jacqui Rees , The Last Shilling: A History of Repatriation in Australia (Carlton: Melbourne University Publishing, 1994), 233 .

26 John Whiting, *Be In It, Mate!* (Dulwich: Veritas Publications, 1969), 111.

27
*Ibid.,* 1.

28 Lloyd and Rees, *op.cit.* (note 25), 326.

29 Lloyd and Rees, *op.cit.* (note 25), 323–8; Garton, *op. cit.* (note 20), 116–7.

30 Parkinson’s Disciple, *op. cit.* (note 18), 209.

31 Parkinson’s Second Disciple, *op. cit.* (note 11), 638.

32 Whiting, *op.cit.* (note 26), 54.

33 Whiting, *op.cit.* (note 26), 57.

34 Whiting, *op.cit.* (note 26), 64.

35 Garton, *op. cit.* (note 20), 87, 88, 146–8; Tyquin, *op.cit* (note 12), 33–8, 48; Richard A.A. Kanaan and Simon C. Wessely , ‘The Origins of Factitious Disorder’, History of the Human Sciences, 23, 2 (2010), 68–85 2054987810.1177/0952695109357128; Joanna Bourke , Dismembering the Male: Men’s Bodies, Britain and the Great War (London: Reaktion Books, 1996), 76–122, 223; Joseph Catton , ‘Malingering: Its Relation to the Doctor’, California State Journal of Medicine, 28, 6 (1920), 220–5PMC159425318738205.

36

Kenneth R. Morel , Differential Diagnosis of Malingering versus Posttraumatic Stress Disorder, (New York: Nova Science Publishers, 2010), 3 .

37 See B. Christopher Frueh, *et al*., ‘Apparent System Overreporting in Combat Veterans Evaluated for PTSD’, *Clinical Psychology Review*, 20, 7 (2000), 853–85; Khodabakhsh Ahmadi, *et al*., ‘Malingering and PTSD: Detecting Malingering and War Related PTSD by Miller Forensic Assessment of Symptoms Test (M-FAST)’, *BMC Psychiatry,* 13,154 (2013), 1–5; Christopher Bass and Peter Halligan, ‘Factitious Disorders 2: Factitious Disorders and Malingering: Challenges for Clinical Assessment and Management’, *The Lancet*, 383 (2014), 1422–32; Brian Andrews, ‘Commentary: Doing the Most Good with the Least Harm in Cases of Suspected Malingering’, *Cambridge Quarterly of Healthcare Ethics*, 27, 4 (2018), 740–2; Derek K. Tracy and Keith J.B. Rix, ‘Malingering Mental Disorders: Clinical Assessment’, *BJPsych Advances*, 23 (2017), 27–35.

38

Daniel S. Goldberg , ‘Doubt & Social Policy: The Long History of Malingering in Modern Welfare States’, The Journal of Law, Medicine & Ethics, 49 (2021), 389–9010.1017/jme.2021.5834665090.

39 American Psychological Association, ‘Malingering’, Z76.5, *Diagnostic and Statistical Manual of Mental Disorders*, 5^th^ ed. (Arlington: American Psychological Association, 2013).

40 Kanaan and Wessely, *op. cit.* (note 35), 77; *Ibid.,* 331.

41

Effie Karageorgos , ‘Mental Illness, Masculinity, and the Australian Soldier: Military Psychiatry from South Africa to the First World War’, Health and History, 20, 2, (2018), 21–2; G.T. Hankins , ‘Clinical and Pathological Notes: Was He a Malingerer?’, Australasian Medical Gazette, 20 November 1902, 572 .

42 Kanaan and Wessely, *op. cit.* (note 35), 75–6; Catton, *op. cit.* (note 35), 221.

43

Yücel Yanikdag , ‘From Cowardice to Illness: Diagnosing Malingering in the Ottoman Great War’, Middle Eastern Studies, 48, 2 (2012), 222 .

44 Parsons, *op. cit.* (note 6), 436, 438, 440.

45 Tyquin, *op. cit.* (note 12), 35.

46

Edgar Jones and Simon Wessely , Shell Shock to PTSD: Military Psychiatry from 1900 to the Gulf War (East Sussex: Psychology Press, 2005), 7–8.

47 Tyquin, *op. cit.* (note 12), 24–5.

48 Tyquin, *op. cit.* (note 12), 34.

49 Bourke, *op. cit.* (note 35), 117.

50

Joy Damousi , Freud in the Antipodes: A Cultural History of Psychoanalysis in Australia (Sydney: UNSW Press, 2005), 37 .

51 Bourke, *op. cit.* (note 35), 115, 118.

52 Garton, *op. cit.* (note 20), 163.

53 Garton, *op. cit.* (note 20), 166.

54 Parkinson’s Apostle, ‘Repatriation Entitlements’, *Medical Journal of Australia*, 1, S9 (1964), 59.

55 See, eg., C.R. Dixon , ‘Courses on Psychology and Students’ Attitudes Toward Mental Illness’, Psychological Reports, 29 (1967), 50 10.2466/pr0.1967.20.1.506037829; Sidney Gelfand and Leonard P. Ullman , ‘Change In Attitudes About Mental Illness Associated With Psychiatric Clerkship Training’, International Journal Of Social Psychiatry, 7 (1961), 292–8; Katherine D. Morris , ‘Behavioral Change: A Concomitant Of Attitude Change In Nursing Students’, Nursing Research, 13, 2 (1964), 132–814153617; W.O. McCormick and George Voineskos , ‘Medical Students’ Views Of Psychiatry: Does Teaching Have Any Effect?’, Journal of the Canadian Psychiatric Association, 238 (1978), 541–710.1177/070674377802300805719588.

56 See Jacob Cohen and E.L. Struening , ‘Opinions about Mental Illness in the Personnel of Two Large Mental Hospitals, Journal of Abnormal and Social Psychology, 64, 5 (1962), 349–601388026710.1037/h0045526; Aikaterini Arvaniti , et al., ‘Health Service Staff’s Attitudes Towards Patients with Mental Illness’, Social Psychiatry and Psychiatric Epidemiology, 44, 8 (2009), 658–651908290510.1007/s00127-008-0481-3.

57

Judith G. Rabkin , ‘Opinions about Mental Illness: A Review of the Literature’, Psychological Bulletin, 77, 3 (1972), 158 10.1037/h00323414554059; Elaine Cumming and John Cumming , Closed Ranks: An Experiment in Mental Health Education (Cambridge: Harvard University Press, 1957); Harold Rosen and James F. Corcoran , ‘The Attitudes of USAF Officers towards Mental Illness: A Comparison with Mental Health Professionals’, Military Medicine, 143, 8 (1978), 570–499702.

58

Robert J. Kirkby and Annie James , ‘Attitudes of Medical Practitioners to Mental Illness’, Australian and New Zealand Journal of Psychiatry, 13, 2 (1979), 166 10.3109/00048677909159132291431.

59
*Ibid.*, 166–7.

60
*Ibid.*, 167.

61 Foucault, *op.cit.* (note 2), 78, 82.

62 Erving Goffman, *op.cit.* (note 2), 4; Szasz, *op. cit.* (note 4), 48–9.

63 Grob, *op. cit.* (note 1), 398.

64 Angrist, *op. cit.* (note 5), 437.

65 Parsons, *op. cit.* (note 6), 436–7.

66 Scheff, *op. cit.* (note 2), 438-–9.

67
*Ibid.,* 441–2.

68 Parkinson’s Second Disciple, *op. cit.* (note 11), 638; Parkinson’s Apostle, *op. cit.* (note 54), 59; R.S. Ellery, ‘A Psychiatric Programme for Peace’, *Medical Journal of Australia*, 1, 14 (1946), 457–65; ‘The Australian Way’, *Smith’s Weekly*, 14 August 1948, 7; Bourke, *op. cit.* (note 35), 110; Whiting, *op.cit.* (note 26), 54.

69 Kirkby and James, *op. cit. (* note 58), 165–8; Angrist, *op. cit.* (note 5), 436.

70 Bates, *op. cit.* (note 1), 549.

71
*Ibid.,* 550.

72

Norman Dain , ‘Critics and Dissenters: Reflections on “Anti-Psychiatry” in the United States’, Journal of the History of the Behavioural Sciences, 25, 1 (1989), 6–7 10.1002/1520-6696(198901)25:1<3::aid-jhbs2300250102>3.0.co;2-g2647837.

73 Crossley, *op. cit.* (note 1), 878; See also Grob, *op. cit.* (note 1), 399.

74 Rabkin, *op. cit.* (note 57), 155.

75 Grob, *op. cit.* (note 1), 411–2; See also Angrist, *op. cit.* (note 5), 436.

76

Dolly MacKinnon and Catharine Coleborne , ‘Introduction: Deinstitutionalisation in Australia and New Zealand’, Health and History, 5, 2 (2003), 8 .

77

E. Cunningham Dax , ‘Psychiatry and Society’, Medical Journal of Australia, 2, 22 (1966), 1018 10.5694/j.1326-5377.1966.tb73786.x5925746; Milton Lewis , Managing Madness: Psychiatry and Society in Australia, 1788–1980 *(Canberra: Australian Government Publishing Services, 1988)*, 75 .

78 Dain, *op. cit.* (note 72), 8.

79 Crossley, *op. cit.* (note 1), 880.

80

Paul Laffey , ‘Antipsychiatry in Australia: Sources for a Social and Intellectual History’, Health and History, 5, 2 (2003), 20 .

81
*Ibid.,* 22.

82 Dunlop and Pols, *op. cit.* (note 1), 97.

83
*Ibid.,* 99; Laffey, *op. cit.* (note 80), 24.

84
*Ibid.,* 92, 98.

85 See E. Cunningham Dax , ‘Psychiatry in Australia’, American Journal of Psychiatry, 124, 2 (1967), 180–6495157210.1176/ajp.124.2.180; McKinnon and Coleborne, *op. cit.* (note 76), 5.

86 Dax, *op. cit.* (note 77), 1019.

87
*Ibid.,* 1019.

88 Dunlop and Pols, *op. cit.* (note 1), 99; Laffey, *op. cit.* (note 80), 24.

89 Laffey, *op. cit.* (note 80), 20–2.

90 Dax, *op. cit.* (note 85), 180–6.

91

Arnold M. Ludwig , ‘The Psychiatrist as Physician’, Journal of the American Medical Association, 234, 6 (1975), 603–41242189; Grob, *op. cit.* (note 1), 410.

92 Lewis, *op. cit.* (note 77), 99.

93 Parkinson’s Second Disciple, *op. cit.* (note 11), 638.

94 Kanaan and Wessely, *op. cit.* (note 35), 75–6; Catton, *op. cit.* (note 35), 221; Yanikdag, *op. cit.* (note 43), 222.

95 Parsons, *op. cit.* (note 6), 436-7.

96 Parkinson’s Second Disciple, *op. cit.* (note 11), 638.

97 Parsons, *op. cit.* (note 6), 436.

98
*Ibid.,* 437.

99
*Ibid.,* 447.

100

C. Gordon Harper , ‘Repatriation Entitlements’, Medical Journal of Australia, 2, 14 (1963), 599 14044362.

101 Ludwig, *op. cit.* (note 91), 603.

102 Damousi, *op. cit.* (note 50), 37; Garton, *op. cit.* (note 20), 163, 166.

103 See American Psychological Association, *op. cit.* (note 39).

104 Grob, *op. cit.* (note 1), 399; Lewis, *op. cit.* (note 77), 99; Parsons, *op. cit.* (note 6), 441.

105 Angrist, *op. cit.* (note 5), 437.

106 Parkinson’s Disciple, *op. cit.* (note 18), 208.

107 Harper, *op. cit.* (note 100), 599.

108 Whiting, *op.cit.* (note 26), 108.

109 Parsons, *op. cit.* (note 6), 447.

110

Howard M. Spiro , ‘When Doctors Get Sick’, Perspectives in Biology and Medicine, 31, 1 (1987), 131 10.1353/pbm.1987.00163696957.

111
*Ibid.,* 437.

112 Whiting, *op.cit.* (note 26), 64.

113

Fiona Reid , Broken Men: Shell Shock, Treatment and Recovery in Britain 1914-1930 (London: Continuum, 2010), 38 ; Joanna Bourke , An Intimate History of Killing (London: Basic Books, 1999), 76, 236; Joshua J. Jackson , et al., ‘Military Training and Personality Trait Development: Does the Military Make the Man, or Does the Man Make the Military?’, Psychological Science, 23, 3 (2012), 276 10.1177/095679761142354522275337.

114

Alistair Thomson , Anzac Memories: Living with the Legend, 2nd edn (Clayton: Monash University Publishing, 2013), 286 .

115

American Psychiatric Association, Diagnostic and Statistical Manual of Mental Disorders: DSM-III (Washington: American Psychological Association, 1980).

116 See Robert J. Lifton , Home from the War: Vietnam Veterans – Neither Victims nor Executioners (New York: Simon & Schuster, 1973); Allan Young , The Harmony of Illusions: Inventing Post-Traumatic Stress Disorder (Princeton: Princeton University Press, 1995), 109–11.

117 Whiting, *op.cit.* (note 26), 54.

118 Parsons, *op. cit.* (note 6), 437.

119 Harper, *op. cit.* (note 100), 599.

120 Parsons, *op. cit.* (note 6), 437.

121 Harper, *op. cit.* (note 100), 599.

122 Parkinson’s Second Disciple, *op. cit.* (note 11), 638.

123 Whiting, *op.cit.* (note 26), 42.

124 Parkinson’s Apostle, *op. cit.* (note 54), 59.

125 Kanaan and Wessely, *op. cit.* (note 35), 78–9.

126
*Ibid.,* 79; Janet Oppenheim , Shattered Nerves: Doctors, Patients and Depression in Victorian England (New York: Oxford University Press, 1991), 144; Reid, *op. cit.* (note 113), 17; Bourke, *op. cit.* (note 35), 112.

127 Sanaullah Khan, ‘Medicine and the Critique of War: Military Psychiatry, Social Classification and the Malingering Patient in Colonial India’*, Medical History*, 66, 1 (2022), 48.

128 Charles E. Rosenberg, ‘The Tyranny of Diagnosis: Specific Entities and Individual Experience’, *The Milbank Quarterly*, 80, 2 (2002), 240.

129 Parkinson’s Apostle, *op. cit.* (note 54), 59; Parkinson’s Disciple, *op. cit.* (note 18), 209.

130 Richard Holmes, *Firing Line* (Middlesex: Penguin, 1987), 244–5.

131 Effie Karageorgos, *Australian Soldiers in South Africa and Vietnam: Words from the Battlefield* (London: Bloomsbury Academic, 2016), 124–5.

132 Tyquin, *op. cit.* (note 12), 105, 129; See also Garton, *op. cit.* (note 20), 95.

133 See, eg., Christina M. Hassija, *et al*., ‘The Influence of Combat and Interpersonal Trauma on PTSD, Depression and Alcohol Misuse in U.S. Gulf War and OEF/OIF Women Veterans’, *Journal of Traumatic Stress*, 25, 2 (2012), 216–9; Michelle L. Kelley, *et al*., ‘Alcohol Use and Trauma Exposure among Male and Female Veterans Before, During, and After Military Service’, *Drug and Alcohol Dependence*, 133, 2 (2013), 615–24.

134 Kanaan and Wessely, *op. cit.* (note 35), 78–9.

135 Whiting, *op.cit.* (note 26), 69.

136 Garton, *op. cit.* (note 20), 148; Bourke, *op. cit.* (note 35), 96.

137 Bourke, *op. cit.* (note 35), 78.

138 Whiting, *op.cit.* (note 26), 67.

139
*Ibid.,* 47.

140 Margaret J. Sargent, ‘Heavy Drinking and Its Relation to Alcohol’, *The Australian and New Zealand Journal of Sociology,* 4, 2 (1968), 146–57.

141 H.M. Southwood, ‘The Psychiatrist and the Public’, *Medical Journal of Australia*, 27 May 1961, 773, 775.

142 B.L. Hennessy, ‘Alcoholism and Drug Dependence: Planning a New Service’, *Australian Journal of Social Issues*, 6, 1 (1971), 18.

143 H. Paul Chalfant and Richard A. Kurtz, ‘Alcoholics and the Sick Role: Assessments by Social Workers’, *Journal of Health and Social Behaviour*, 12, 1 (1971), 66–7.

144 H. S. Abram and W. F. McCourt, ‘Interaction of Physicians with Emergency Ward Alcoholic Patients’, *Quarterly Journal of Studies on Alcohol*, 25 (1964), 679.

145 Stephen T. Casper, ‘Punch-Drunk Slugnuts: Violence and the Vernacular History of Disease’, *Isis*, 113, 2 (2022), 269.

146 Roger Jeffery, ‘Normal Rubbish: Deviant Patients in Casualty Departments’, *Sociology of Health and Illness*, 1, 1 (1979), 90–107; P.M. Strong, ‘Doctors and Dirty Work – the Case of Alcoholism’, *Sociology of Health and Illness*, 2, 1 (1980), 24–47.

147 Strong, *op. cit.* (note 146), 29.

148 Parsons, *op. cit.* (note 6), 436.

149 Jeffery, *op. cit.* (note 146), 97, 100.

150 Joel T. Braslow, ‘The Influence of a Biological Therapy on Physicians’ Narratives and Interrogations: The Case of General Paralysis of the Insane and Malaria Fever Therapy, 1910–1950’, *Bulletin of the History of Medicine*, 70, 4 (1996), 594, 597.

151 Jack H. Mendelson, *et al*., ‘Physicians Attitudes Towards Alcoholic Patients’, *Archives of General Psychiatry*, 11, 4 (1964), 398.

152 Patricia A. Sowa and Henry S. Cutter, ‘Attitudes of Hospital Staff toward Alcoholics and Drug Addicts’, *Quarterly Journal of Studies on Alcohol*, 35, 1-A (1974), 214.

153 Parsons, *op. cit.* (note 6), 437.

154 Mendelson *et al*., *op. cit.* (note 151), 392.

155 Jeffery, *op. cit.* (note 146), 101.

156 Parkinson’s Second Disciple, *op. cit.* (note 7), 638.

157 Jeffery, *op. cit.* (note 137), 100.

158 Australian Institute of Health and Welfare, *Incidence of Suicide among Serving and Ex-serving Australian Defence Force Personnel 2001–2015: In Brief Summary Report* (Canberra: Australian Institute of Health and Welfare, 2017).

159 American Psychiatric Association, *op. cit.* (note 115).

160 Royal Commission into Defence and Veteran Suicide, Commonwealth of Australia, https://defenceveteransuicide.royalcommission.gov.au/.

161 Royal Commission into Defence and Veteran Suicide, *Overview of roundtable discussions – November and December 2021*, 4 July 2022, https://defenceveteransuicide.royalcommission.gov.au/publications/overview-roundtable-discussions-november-and-december-2021.

162 Royal Commission into Defence and Veteran Suicide, *Interim Report* (Canberra: Commonwealth of Australia, 2022), 36, 37.

163 Whiting, *op.cit.* (note 26), 64.

